# Detection of *Wolbachia* in field-collected *Aedes aegypti* mosquitoes in metropolitan Manila, Philippines

**DOI:** 10.1186/s13071-019-3629-y

**Published:** 2019-07-24

**Authors:** Thaddeus M. Carvajal, Kazuki Hashimoto, Reza Kurniawan Harnandika, Divina M Amalin, Kozo Watanabe

**Affiliations:** 1 Department of Civil and Environmental Engineering, Ehime University, Matsuyama, Japan; 2 Biology Department, De La Salle University, Taft Avenue, Manila, Philippines; 3 Biological Control Research Unit, Center for Natural Science and Environmental Research, De La Salle University, Taft Avenue, Manila, Philippines

**Keywords:** *Wolbachia*, Metropolitan Manila, Dengue, *Ae. aegypti*

## Abstract

**Background:**

Recent reports reveal the presence of *Wolbachia* in *Ae. aegypti*. Our study presents additional support for *Wolbachia* infection in *Ae. aegypti* by screening field-collected adult mosquitoes using two *Wolbachia*-specific molecular makers.

**Methods:**

A total of 672 *Ae. aegypti* adult mosquitoes were collected from May 2014 to January 2015 in Metropolitan Manila. Each individual sample was processed and screened for the presence of *Wolbachia* by selected markers, *Wolbachia*-specific *16S* rDNA and its surface protein (*wsp*), under optimized PCR conditions and sequenced.

**Results:**

Totals of 113 (16.8%) and 89 (13.2%) individual mosquito samples were determined to be infected with *Wolbachia* using the *wsp* and *16S* rDNA markers, respectively. The *Ae. aegpyti wsp* sample sequences were similar or identical to five known *Wolbachia* strains belonging to supergroups A and B while the majority of *16S* rDNA sample sequences were similar to strains belonging to supergroup B. Overall, 80 (11.90%) individual mosquito samples showed positive amplifications in both markers and 69% showed congruence in supergroup identification (supergroup B).

**Conclusions:**

By utilizing two *Wolbachia*-specific molecular makers, our study demonstrated the presence of *Wolbachia* from individual *Ae. aegypti* samples. Our results showed a low *Wolbachia* infection rate and inferred the detected strains belong to either supergroups A and B.

**Electronic supplementary material:**

The online version of this article (10.1186/s13071-019-3629-y) contains supplementary material, which is available to authorized users.

## Background

*Wolbachia* is a naturally occurring endosymbiont which can be maternally inherited and cause different reproductive alterations in its host to increase their transmission to the next generation [1–3]. In insects, it is estimated to be naturally present in 60–65% of known species [4]. Currently, there are 17 identified major clades or supergroups (A–Q), the majority of which are known to infect arthropods such as insects, arachnids and crustaceans [5]. The pathogenic effects of *Wolbachia* in its host are well-studied and determined to cause sperm-egg incompatibility, parthenogenesis, cytoplasmic incompatibility and feminization [2, 6]. Therefore, utilizing these effects in medically-important mosquito vectors, has resulted in significant progress in the past two decades.

Previous studies claimed that medically important mosquitoes such as *Culex* spp., *Mansonia* spp., and *Aedes albopictus* were naturally infected with *Wolbachia*, whereas *Ae. aegypti* was not [7–12]. A more recent global survey from 27 countries also established the absence of *Wolbachia* in *Ae. aegypti* [13]. However, numerous studies now contradict this claim and present evidence of natural *Wolbachia* infection in *Ae. aegypti*, including recent studies from Malaysia [14], India [15] and the USA [16]. The first ever report came from *Ae. aegypti* larval samples in Malaysia [14]; however, the sample size was too small (*n *= 16) to affirm such findings. Afterwards, metabarcoding studies by examining bacterial communities in the midgut of *Ae. aegypti* in the USA and Thailand reported a low presence of *Wolbachia* sequences [17, 18]. In 2019, evidence of natural *Wolbachia* infection in *Ae. aegypti* from India was presented, based on amplification of *Wolbachia*-specific *16S* rRNA, *wsp* and *ftsZ* molecular markers [15]. This was followed by a report of *Wolbachia* presence *Ae. aegypti* populations in the USA, specifically from the states of New Mexico and Florida, using *16S* rDNA, *gatB*, *ftsZ* and strain-specific (phosphoesterase and diaminopimelate epimerase) markers [16]. Both demonstrated the persistence of the endosymbiont across the developmental stages of *Ae. aegypti* through cytological examination and molecular detection. This clearly illustrates that the infection of *Wolbachia* in *Ae. aegypti* appears common than previously recognized.

This report provides additional support to the presence of *Wolbachia* in field-collected *Ae. aegypti* adult mosquitoes using *Wolbachia*-specific *16S* rDNA and its surface protein (*wsp*). In comparison to previous studies, we conducted a large sampling of *Ae. aegypti* mosquitoes (*n *= 672) in a microgeographical area in order to discern the spatial distribution of *Wolbachia-*infected mosquitoes at the city scale and also to more accurately understand the infection rate in a natural *Ae. aegypti* population. Furthermore, our results focus primarily on the global phylogeny of *Wolbachia* strains within *Ae. aegypti*. In previous studies [15, 16], the *Wolbachia* strain isolated belonged to supergroup B or homologous to the strain from *Ae. albopictus*. Not only do our results conform to these previous findings, but they also reveal other prospective *Wolbachia* strains (e.g. supergroup A) infecting this mosquito.

## Methods

### Study area and mosquito collection

The study area was the National Capital Region of the Philippines, also known as Metropolitan Manila. Located on the eastern shore of Manila Bay in southwestern Luzon Island (14°35′58.2432″N, 121°59′3.1992″E), it is considered to be one of the most highly urbanized and densely populated areas in the Philippines. Dengue disease is endemic in this region where it accounted for 15–25% of the total number of reported dengue cases annually in the period 2009–2014 [19].

**Fig. 1:**
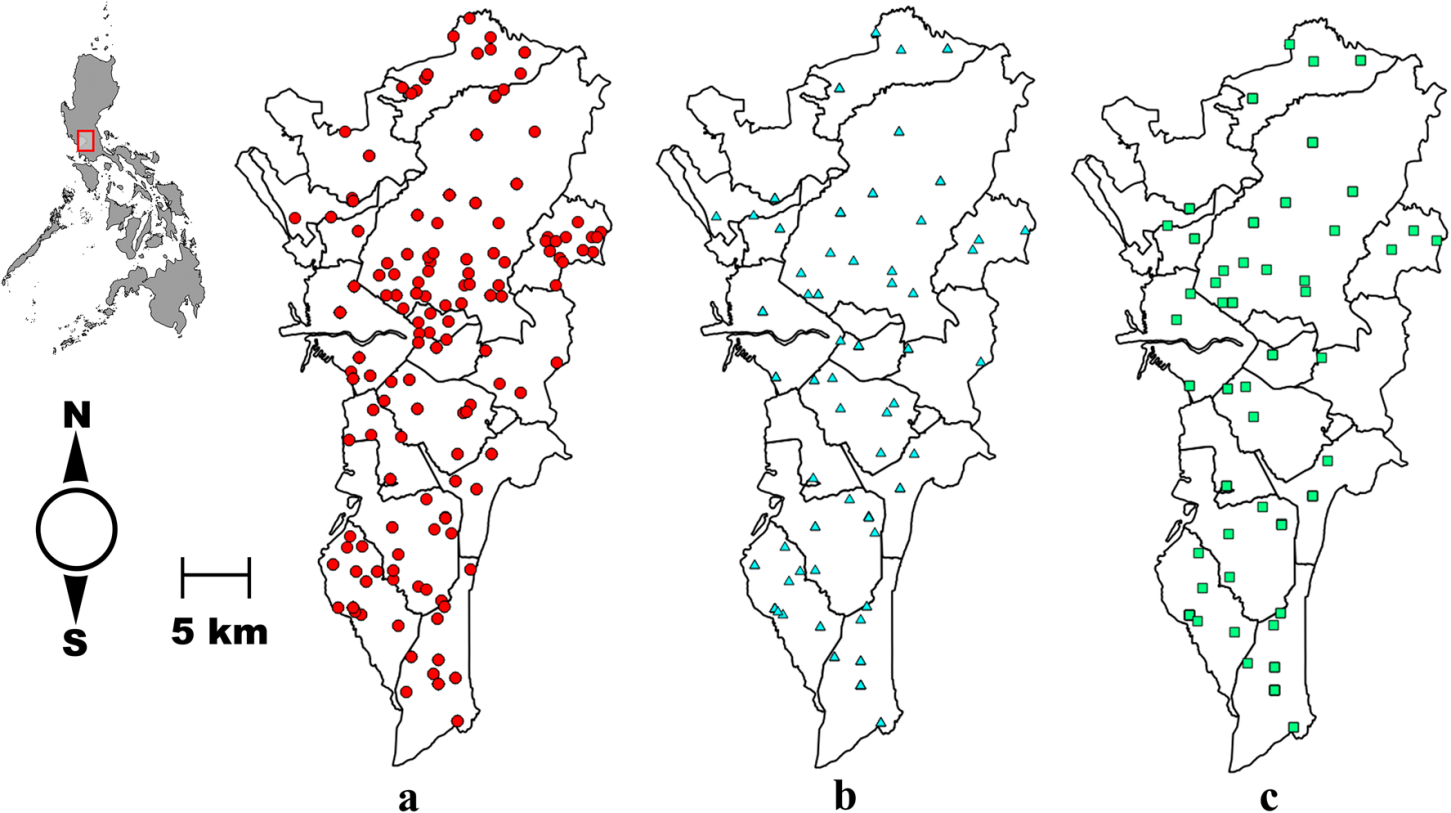
**a** Spatial distribution of the sampling sites (*n *= 138) for collecting adult *Ae. aegypti*. *Wolbachia*-positive sampling sites (circles) based on **b** *wsp* (triangles) and **c** *16S* rDNA (squares). Details of the number of *Wolbachia*-positive mosquitoes per sampling site are provided in Additional file 1: Table S1

Adult mosquito samples were collected using a commercial branded mosquito UV-light trap (MosquitoTrap®; Jocanima Corporation, Las Piñas City, Philippines) installed in the outdoor premises of 138 residential households (sampling sites) from May 2014 to January 2015 (Fig. 1a). Collected mosquito samples were then sorted and identified as *Ae. aegypti* using available keys [20]. This was then placed in a tube with 99.5% ethanol for preservation. In total, 672 *Ae. aegypti* adult mosquito samples were collected, identified, labeled (see Additional file 1: Table S1) and stored at − 20 °C for subsequent processing.

### DNA extraction, PCR amplification and sequencing

Total genomic DNA of each mosquito individual was extracted using a Blood and Tissue DNEasy Kit© (Qiagen, Hilden, Germany) following a modified protocol [21]. Our study used two molecular markers for detecting *Wolbachia* infection, namely *wsp* [22] and *16S* rDNA [23]. The primer sequences were *wsp* 81F (5′-TGG TCC AAT AAG TGA TGA AGA AAC-3′) and *wsp* 691R (5′-AAA AAT TAA ACG CTA CTC CA-3′) for the *wsp* marker and the *16S Wolbachia*-specific primers were *WolbF* (5′-GAA GAT AAT GAC GGT ACT CAC-3′) and *Wspecr* (5′-AGC TTC GAG TGA AAC CAA TTC-3′).

For the *wsp* gene amplification, we followed the standard *wsp* protocol [11] where the suggested annealing temperature and number of cycles were 55 °C and 30 cycles, respectively. To conduct an individual-based detection, we initially performed this protocol using *Culex quinquefasciatus* as our positive control. Certain modifications were made in the standard protocol based on these results. The annealing temperature was increased to 57 °C and the number of cycles was increased to 35 cycles. This initial modified protocol was performed in individual *Ae. aegypti* samples where it yielded positive faint bands. As a result, we modified the protocol again, setting the annealing temperature at 59 °C with 40 cycles, and adding 10% DMSO (Sigma-Aldrich, St. Louis, Missouri, USA). This led to desirable results necessary for sequencing. In the end, a 10 μl final reaction volume was used consisting of 10× buffer (TaKaRa, Shiga, Japan), 25 mM MgCl_2_, 10 mM of each dNTPs, 10 μM forward and reverse primers, 10% DMSO (Sigma-Aldrich) and 5.0 U/μl of *Taq* DNA polymerase (TaKaRa). The final thermal profile was as follows: initial denaturation at 95 °C for 3 min; 40 cycles of denaturation at 95 °C for 1 min, annealing at 59 °C for 1 min and extension at 72 °C for 1 min; final extension at 72 °C for 3 min.

For the *16S* rDNA gene amplification, we used a 10 μl final reaction volume consisting of 10× buffer (TaKaRa), 25 mM MgCl_2_, 10 mM of each dNTPs, 10 μM forward and reverse primers, 10% DMSO (Sigma-Aldrich) and 5.0U/μl of *Taq* DNA polymerase (TaKaRa). Thermal profiles followed the protocol of Simões et al. [23]: initial denaturation at 95 °C for 2 min; two cycles of denaturation at 95 °C for 2 min, annealing at 60 °C for 1 min and extension at 72 °C for 1 min; 35 cycles of denaturation at 95 °C for 30 s, annealing at 60 °C for 1 min and extension at 72 °C for 45 s; final extension at 72 °C for 10 min.

All PCR amplification experiments included positive and negative controls. The positive control was a *Wolbachia*-infected *Cx. quinquefasciatus* sample while the negative control was water. The product size of each molecular marker was checked through 1.5% agarose gel electrophoresis set at 100 V for 30 min. The size of the amplified *wsp* gene is approximately 610 bp while the *16S* rDNA gene is approximately 850 bp. The PCR amplification process underwent two replicates to validate the results (see Additional file 1: Table S1). A third screening was performed for selected individual samples that had inconsistent results based on the two prior replicates. The criteria set to confirm *Wolbachia* infection were based on two successful amplifications of the molecular markers. Furthermore, individual samples that met this criterion were subjected for sequencing through Eurofins Genomics, Tokyo.

### Identity of *Wolbachia* strains and their positions in phylogroups

All sequences were subjected to the Nucleotide Basic Local Alignment Search Tool (BLAST) and compared to deposited *Wolbachia* sequences in GenBank. The selected sequences of *Wolbachia* strains (Table 1) and those obtained in the study then underwent multiple alignment using Clustal W in MEGA 6 [24]. After editing, the final lengths used for phylogenetic inference analyses were 398 and 721 bp for *wsp* and *16S* rDNA, respectively. The identities and relationships of the *Wolbachia* strains obtained in our study were determined by performing Bayesian inference analysis using PhyML v.3.0 software with 1000 bootstrap replicates [25]. Smart Model Selection [26] was also utilized to set the parameters for *wsp* as GTR+G (number of estimated parameters k= 232, Akaike information criterion (AIC)= 4897.31702) and *16S* rDNA as GTR+G+1 (number of estimated parameters k= 207, AIC= 5332.88688). All newly generated sequences were submitted to the GenBank database with accession numbers MN046588–MN046789.

**Table 1:** Representative *Wolbachia* type sequences from different insect hosts in *wsp* and *16S* rDNA molecular markers

Molecular marker	Host	*Wolbachia* supergroup	GenBank ID
*wsp*	*Drosophila melanogaster*	A	AF020072
	*Aedes albopictus*	A	AF020058
	*Glossina morsitans*	A	AF020079
	*Drosophila simulans* (Riverside)	A	AF020070
	*Muscidifurax uniraptor*	A	AF020071
	*Phlebotomus papatasi*	A	AF020082
	*Glossina austeni*	A	AF020077
	*Culex pipiens*	B	AF020061
	*Culex quinquefasciatus*	B	AF020060
	*Aedes albopictus*	B	AF020059
	*Aedes aegypti*	B	MF999264
	*Ephestia cautella*	B	AF020076
	*Dirofilaria immitis*	C (outgroup)	AJ252062
*16S* rDNA	*Nasonia longicornis*	A	M84691
	*Muscidifurax uniraptor*	A	L02882
	*Aedes albopictus*	B	KX155506
	*Aedes aegypti*	B	MF999263
	*Culex pipiens*	B	X61768
	*Nasonia vitripennis*	B	M84686
	*Onchocera volvulus*	C	AF069069
	*Dirofilaria immitis*	C	Z49261
	*Litomosa westi*	D	AJ548801
	*Folsomia candida*	E	AF179630
	*Mansonella ozzardi*	F	AJ279034
	*Dipetalonema gracile*	J	AJ548802
	*Rickettsia* sp.	Outgroup	U11021

### Statistical analysis

A Clark-Evans test was performed to determine whether the spatial distribution of *Wolbachia*-positive mosquito samples from each molecular marker had a pattern of complete spatial randomness. The test uses the aggregation index (*R*), where a value > 1 suggests an ordered distribution and a value < 1 suggests clustering. This analysis was performed using R v.3.3.5 (package *spatstat*) [27].

## Results

### Detection of *Wolbachia* through *wsp* and its phylogeny

**Fig. 2:**
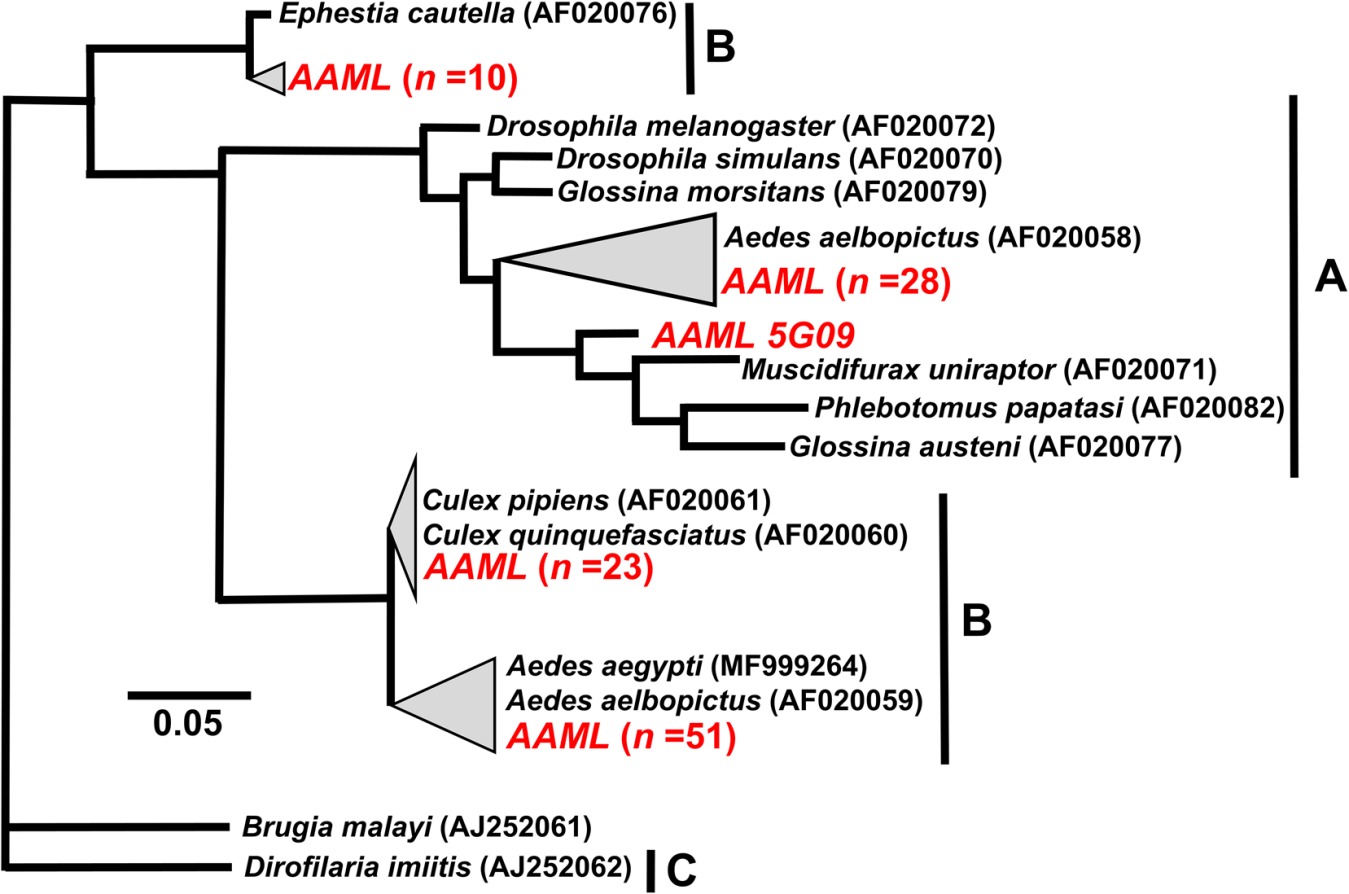
Phylogenic analysis based on *wsp* gene. The alignment was analyzed in PhyML. Sample sequences of *Ae.aegypti* collected in Metropolitan Manila are in red, labeled as AAML (***A****e.* ***a****egypti* **M**etropolitan Mani**L**a) and alphanumeric values indicate the unique code assigned to each *Ae. aegypti* individual sample. Merging (gray triangles) of sample and representative *Wolbachia* sequences was done to show degree of similarity (98–100%). Supergroups are indicated as **A**–**C** depending on the representative sequences used. The phylogenetic trees are re-drawn for better visualization; the expanded version is provided in Additional file 2: Figure S1. Please refer to Table 1 for the *Wolbachia* type sequences (ingroup and outgroup) for both markers

From a total of 672 adult *Ae. aegypti* screened using the *wsp* marker, 113 (16.8%) individual adult mosquito samples were positive for *Wolbachia* infection (Table 2) based on the study criteria (see Methods). Other than the positive individual adult mosquito samples, there were also 17 individual samples that produced one successful *wsp* amplification; however, these were excluded in reporting the prevalence and further analysis. The female/male ratio was 0.82 (Table 2). All sequenced amplicons resulted in a high degree of similarity (> 98.0%) with the *wsp* sequences in GenBank. The spatial distribution showed that 60 (43.0%) sampling sites (Fig. 1b) contained *Wolbachia* infected mosquitoes. Positive sampling sites had prevalence rates ranging between 7.69–100%. Further analysis showed that the distribution of *wsp*-positive mosquito samples was significantly clustered (*R *= 0.003, *P* < 0.001). The *wsp* phylogeny indicated that majority of the sequences belong to supergroup B (*n *= 84) while the remaining were in supergroup A (*n *= 29) (Fig. 2 and Additional file 2: Figure S1). Based on descending order of sample sizes, sample sequences from supergroup B were identical (> 99.0%) to *Wolbachia* type strains from selected hosts such as *Ae. albopictus* (*wAlbB*), *Ae*. *aegypti* (*wAegB)* (*n *= 51), *Cx. quinquefasciatus*, *Cx. pipiens* (*wPip*) (*n *= 23) and *Ephestia cautella* (*wCau*) (*n *= 10). The sample sequences from supergroup A were either similar to (98.0–99.0%) (*n *= 8) or identical (> 99.0%) (*n *= 21) with the *Wolbachia* strain (*wAlbA*) found in *Ae. albopictus*.

**Table 2:** Summary *of wsp* and *16S rDNA* detection results in *Ae. aegypti*

Molecular marker	No. of individuals detected (%) (*n *= 672)	Female (*n *= 379)	Male (*n *= 293)	Female/male ratio
*wsp*	113 (16.82)	52	61	0.82
*16S* rDNA	89 (13.24)	41	48	0.85
*wsp* +*16S* rDNA	80 (11.90)	36	44	0.82

### Detection of *Wolbachia* through *16S* rDNA and its phylogeny

**Fig. 3:**
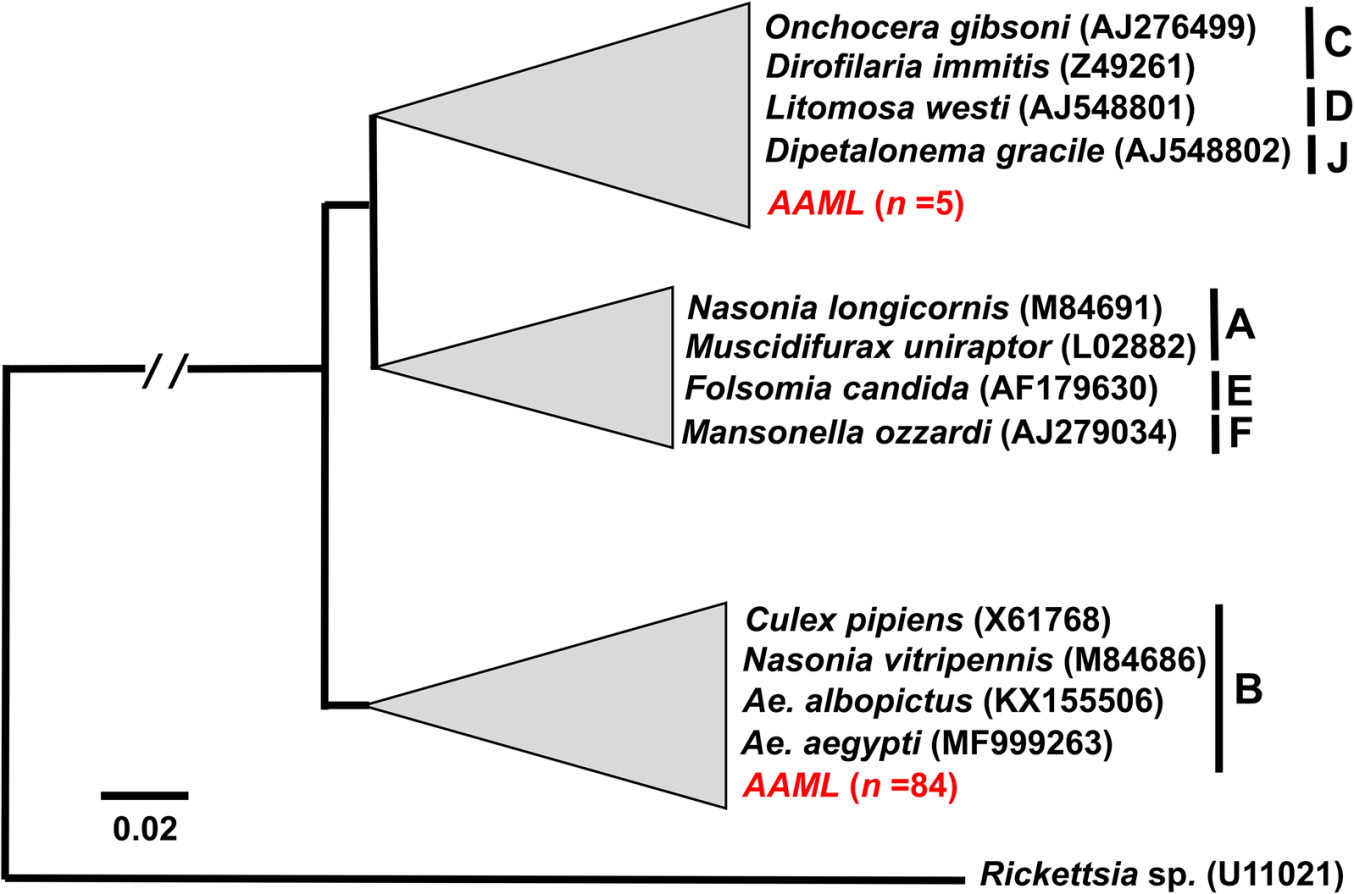
Phylogenic analysis based on *16S* rDNA. The alignment was analyzed in PhyML. Sample sequences of *Ae.aegypti* collected in Metropolitan Manila are in red, labeled as AAML (***A****e.* ***a****egypti* **M**etropolitan Mani**L**a) and alphanumeric values indicate the unique code assigned to each *Ae. aegypti* individual sample. Merging (gray triangles) of sample and representative *Wolbachia* sequences was done to show degree of similarity (98–100%). Supergroups are indicated as A to J depending on the representative sequences used. The phylogenetic trees are re-drawn for better visualization; the expanded version is provided in Additional file 3: Figure S2. Please refer to Table 1 for the *Wolbachia* type sequences (ingroup and outgroup) for both markers

For the *16S* rDNA, 89 (13.2%) individual adult mosquito samples were infected with *Wolbachia* (Table 2) based on the study criteria. In addition to these, 20 individual mosquito samples generated one successful *16S* rDNA amplification, but were excluded in reporting the prevalence and further analysis. The female/male ratio was 0.85 (Table 2). Fifty (36.0%) sampling sites (Fig. 1c) contained *Wolbachia*-infected mosquitoes. Positive sampling sites had prevalence rates ranging from 3.9 to 100%. The distribution of *16S* rDNA-positive individuals was revealed to be clustered or aggregated (*R *= 0.001, *P* < 0.001). All sequenced amplicons resulted in a high degree of similarity (> 98%) with *16S* rDNA *Wolbachia* sequences in GenBank. Nearly all *16S* rDNA sample sequences (*n *= 84) (Fig. 3, Additional file 3: Figure S2) belonged to supergroup B. Only one sample sequence was identical to the endosymbiont found in *Nasonia vitripennis* while 27 sample sequences were identical to *Wolbachia* isolated from *Ae. aegypti*. The remaining sample sequences from supergroup B were 99% similar from selected hosts of the supergroup. Five sample sequences were grouped together with *Wolbachia* hosts in supergroups C, D and J. Only one sample sequence was highly similar (> 99%) to *Dirofilaria immitis* while the remaining were 98–99% similar to the selected hosts of the supergroup.

### Comparison of *16S* rDNA and *wsp* for *Wolbachia* detection and phylogeny

A total of 80 (11.90%) individual samples yielded positive amplification in both markers (Table 2). In the *wsp* positive samples (*n *= 113), 80 had two successful amplifications of the *16S* rDNA amplification while 27 had only one successful *16S* rDNA amplification and the remaining 6 had no successful *16S* rDNA amplification. For the *16S* rDNA positive samples (*n *= 89), there were 80 individuals with two successful *wsp* amplification, while 9 had only one successful *wsp* amplification. We then focused on the supergroup classification of the 80 individual samples based on the *wsp* and *16S* rDNA phylogeny. It was found that 55 samples (69%) belonged to supergroup B while the remaining 25 samples (31%) showed disparity. In certain instances, *wsp* identified an individual sample as supergroup A, but *16S* rDNA revealed it as either supergroup B, C or J.

## Discussion

In our study, we found a low infection rate (11%) of *Wolbachia* in the *Ae. aegypti* population studied. This finding coincides with the low infection rate reported in Florida [16]; however, a higher infection rate (> 50%) was observed in *Ae. aegypti* populations in Malaysia [14] and New Mexico [16]. It has been established that it is common to see varying *Wolbachia* infection rates of the same insect host from different geographical locations such as that observed in *Cx. quienquefasciatus* [28, 29] and *Cx. pipiens* [30–32]. The variation of infection rates could be driven by either genetic or environmental factors [16]. *Wolbachia* density in *Ae. albopictus* tends to decrease if exposed to increasing temperatures [33]. Removal of the endosymbiont from its host could be achieved by exposure to heat treatment or even antibiotics [34, 35]. The observed low infection rate could be attributed to the low density of the endosymbiont in *Ae. aegypti*. This is further supported by metabarcoding studies which yielded a low number (2–10) [16–18] of sequence reads in the midgut of *Ae. aegpyti* which indicate a low probable density of the endosymbiont. Although our study did not measure the actual density, a 40-cycle PCR amplification procedure or long PCR run [36] was needed to amplify and confirm a positive infection of the endosymbiont in our *Ae. aegypti* samples.

Based on the results of our phylogenetic analysis, the *Wolbachia* strains found in our sampled *Ae. aegypti* belong to supergroups A and B. Both *wsp* and *16S* rDNA phylogeny showed that the majority of the individual samples belong to supergroup B while a small number of individual samples belong to supergroup A (based on *wsp*). The same observation has been reported in previous studies [14–18]. Detecting different *Wolbachia* strains in a single mosquito species is relatively common, especially in medically-important mosquitoes such as *Ae. albopictus* [22, 37], *An. gambiae* [38] and other insect host species (e.g. *Drosophila* species [37]). Dipterans, especially mosquitoes, are commonly infected by *Wolbachia* strains from supergroups A and B. They have been shown to cause parasitism towards the insect host by producing phenotypic effects such as cytoplasmic incompatibility, male killing and feminization [39]. However, it remains unclear whether the identified *Wolbachia* strains in *Ae. aegypti* induce these phenotypic effects. Further studies are needed to confirm the pathogenic impact of this endosymbiont to the mosquito vector. It is also important to determine whether these identified *Wolbachia* strains could inhibit the replication of arboviruses such as dengue, rendering *Ae. aegypti* a less effective vector. A few individual samples (*n *= 5) were shown to be similar to *Wolbachia* strains found in supergroups C, D and J based on *16S* rDNA. It is likely that our *16S* rDNA amplified the *Wolbachia* strain found in the roundworm, *Dirofilaria immitis*, a parasitic nematode that *Ae. aegpyti* mosquitoes also carry and transmit to certain mammals, such as dogs [40]. This observation was also reported in one of the metabarcoding studies that showed sequences of *Wolbachia* from *Dirofilaria immitis*. However, when these *16S* rDNA results were compared to the *wsp* results in our study, it showed the *Wolbachia wsp* sample sequence of the same mosquito individuals belonged to supergroup B. We assume that this discordance may stem from the different mutation rates of the markers used. *16S* rDNA is known to be a conserved gene; however, in some instances the typing system of this marker has been shown to be insufficient in establishing correct supergroup classification due to its low evolutionary rate [41]. This indicates a potential drawback of *16S* rDNA as a less robust marker in estimating intraspecific phylogenetic relationship among *Wolbachia* supergroup members.

Previous studies reported the non-detection of *Wolbachia* in *Ae. aegypti* which is in contrast with both our results and with recent *Wolbachia* detection reports in this mosquito vector from India, Malaysia and the USA [14–16]. The reasons for these contrasting observations could be attributed to the following: (i) individual vis-à-vis pooled detection assays; (ii) procedural modifications; and (iii) sample size. Kulkarni et al. [16] emphasized that individual screening is more suitable in detecting *Wolbachia* in *Ae. aegypti* due to the low density load of the endosymbiont in the mosquito vector. They tested the sensitivity of a PCR assay containing a pool of 19 *Wolbachia-*negative individuals and one positive individual each from *Ae. albopictus* and *Ae. aegypti*. The results showed that *Wolbachia* could be detected in a pool containing DNA from a single positive *Ae. albopictus* but not in a pool containing DNA from a single *Ae. aegypti* specimen. These results strengthen the notion that *Wolbachia* prevalence studies in *Ae. aegypti* could be inaccurate due to pooled detection assays. Non-optimal DNA amplification and extraction methods could also compromise the results of detection assays. This was demonstrated and emphasized in studies detecting *Wolbachia* from *An*. *gambiae* [38, 42, 43]. Our study conducted a longer PCR run (i.e. 40 PCR cycles) as compared to the general protocol just to produce satisfactory positive bands in gel electrophoresis. Due to the varying and potentially low infection rate of *Ae. aegypti* observed in different studies including ours, a larger sample size would provide a more accurate estimate of its prevalence. Assessing several reports of the prevalence of *Wolbachia* published before 2017 showed that the greatest number of individuals screened was 119 [11], resulting in the non-detection of the endosymbiont. This sample size is very small compared to our study which screened 672 individual mosquitoes, while similar studies screened 288–554 mosquito individuals. In a more recent report in 2018, 2663 *Ae. aegypti* mosquitoes were screened from 27 countries, yet the results showed no presence of *Wolbachia* in these samples [13] However, the screening of *Wolbachia* in this global survey was done in pools consisting up to 20 individuals, which may have compromised the detection of this endosymbiont in *Ae. aegypti* as previously discussed by Kulkarni et al. [16].

Our study acknowledges the uncertainties associated with conventional PCR detection such as high false positive detection rates. With this in mind, we were cautious in affirming a positive infection in each *Ae. aegypti* adult sample. First, the selection of markers is based on the study of Simoes et al. [23] which produced low false positive and false negative rates. Secondly, our study performed replications with strict criteria for a successful *Wolbachia* infection in each mosquito sample. Additionally, a similar study conducted in the USA [16] identified individual mosquito samples with *Wolbachia* by conducting two rounds of PCR detection. Although there are several genetic markers (e.g. MLST genes) and techniques (e.g. IFA, FISH or whole-genome sequencing) available, this short report is limited in presenting the possible detection of *Wolbachia* using a conventional PCR-based approach. We are conducting similar experiments (see recent studies [15, 16]) to substantiate the infection status of *Wolbachia* in this mosquito vector. Mosquito colonies are now being reared in order to establish the maternal inheritance and persistence of *Wolbachia* infection through different mosquito developmental stages and generations.

## Conclusions

The study demonstrated the detection of *Wolbachia* from field-collected *Ae. aegypti* in Metropolitan Manila, Philippines. Totals of 113 (16.8%) and 89 (13.2%) individual mosquito samples were determined to be infected with *Wolbachia* using the *wsp* and *16S* rDNA markers, respectively. Overall, 80 (11.90%) individual mosquito samples showed positive amplifications in both markers, indicating a low infection rate. Our study supports previous studies that the potential *Wolbachia* strain in *Ae. aegypti* belongs to supergroup B. In addition, other *Wolbachia* strains (e.g. supergroup A) could potentially infect this mosquito vector.

## Additional files

**Additional file 1: Table S1.** Demographic profile (sex, sampling site code, location), detection status (*wsp* and *16S* rDNA) of all individual adult *Aedes aegypti* mosquitoes used in the study and their supergroup classification.

**Additional file 2: Figure S1.** Complete *wsp* phylogeny of *Wolbachia* from *Ae. aegypti* (*n *= 113). The alignment was analyzed in the program PHYML and *Wolbachia* host *Dirofilaria immitis* and *Brugia malayi* were selected as outgroups. All sample sequences are indicated as red dots. The condensed version of this tree is presented in Fig. 2.

**Additional file 3: Figure S2.** Complete *16S* rDNA phylogeny of *Wolbachia* from *Ae. aegypti* (*n *= 85). The alignment was analyzed in the program PHYML and *Rickettsia* sp. was selected as an outgroup. All sample sequences are indicated as red dots. The condensed version of this tree is presented in Fig. 3.

## Data Availability

All data generated or analyzed during this study are included in this published article and its additional files. All newly generated sequences are available in the GenBank database under the Accession Numbers MN046588–MN046789.
