# Supplementary figures and images for: Detection of Wolbachia in field-collected Aedes aegypti mosquitoes in metropolitan Manila, Philippines
Source: Parasit Vectors. 2019 Jul 24;12:361. doi: 10.1186/s13071-019-3629-y (PMC6657204; doi:10.1186/s13071-019-3629-y)

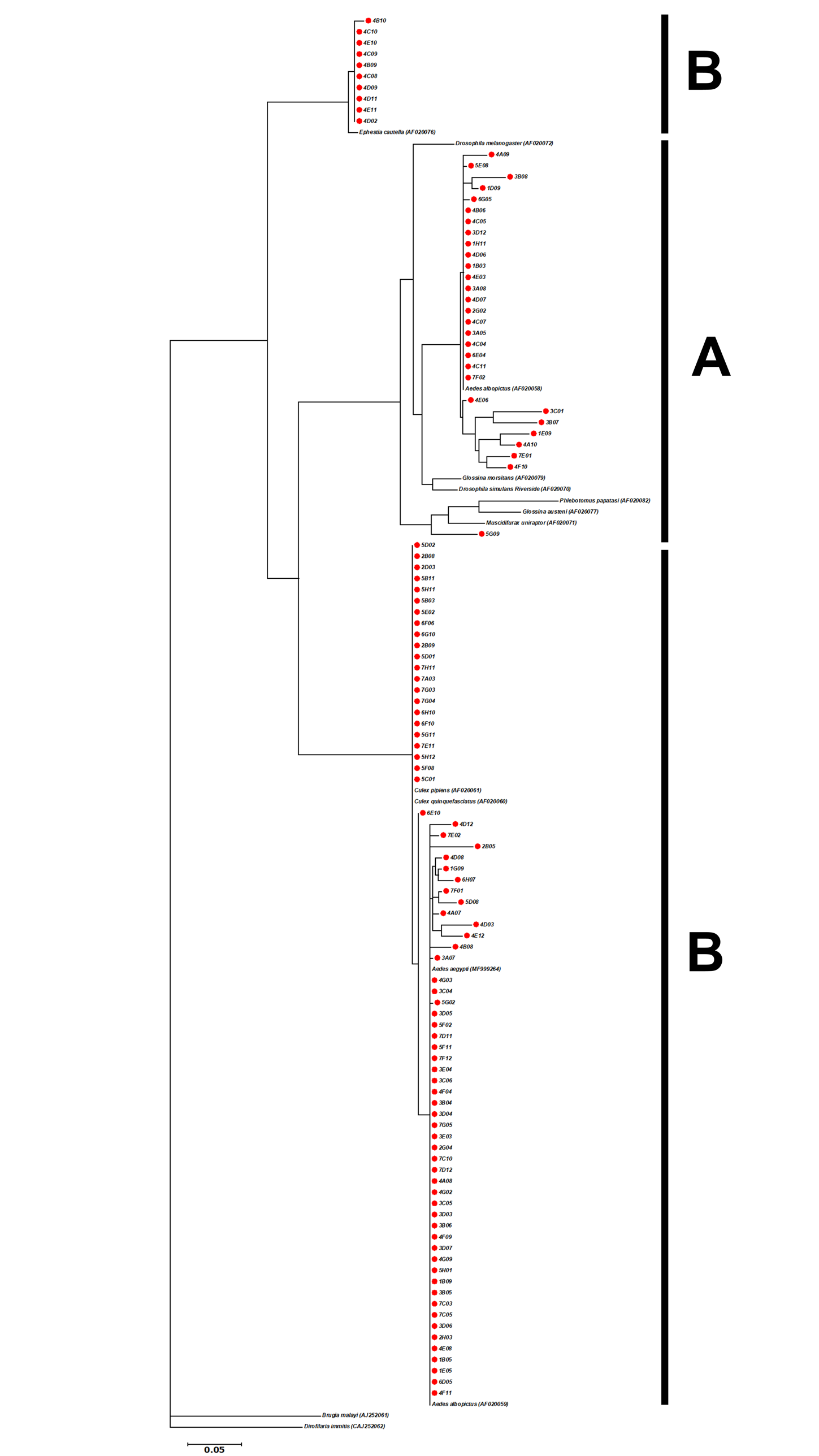

Supplement: Supplementary file 2 — Additional file 2: Figure S1. Complete wsp phylogeny of Wolbachia from Ae. aegypti (n = 113). The alignment was analyzed in the program PHYML and Wolbachia host Dirofilaria immitis and Brugia malayi were selected as outgroups. All sample sequences are indicated as red dots. The condensed version of this tree is presented in Fig. 2. [file 13071_2019_3629_MOESM2_ESM.tif]

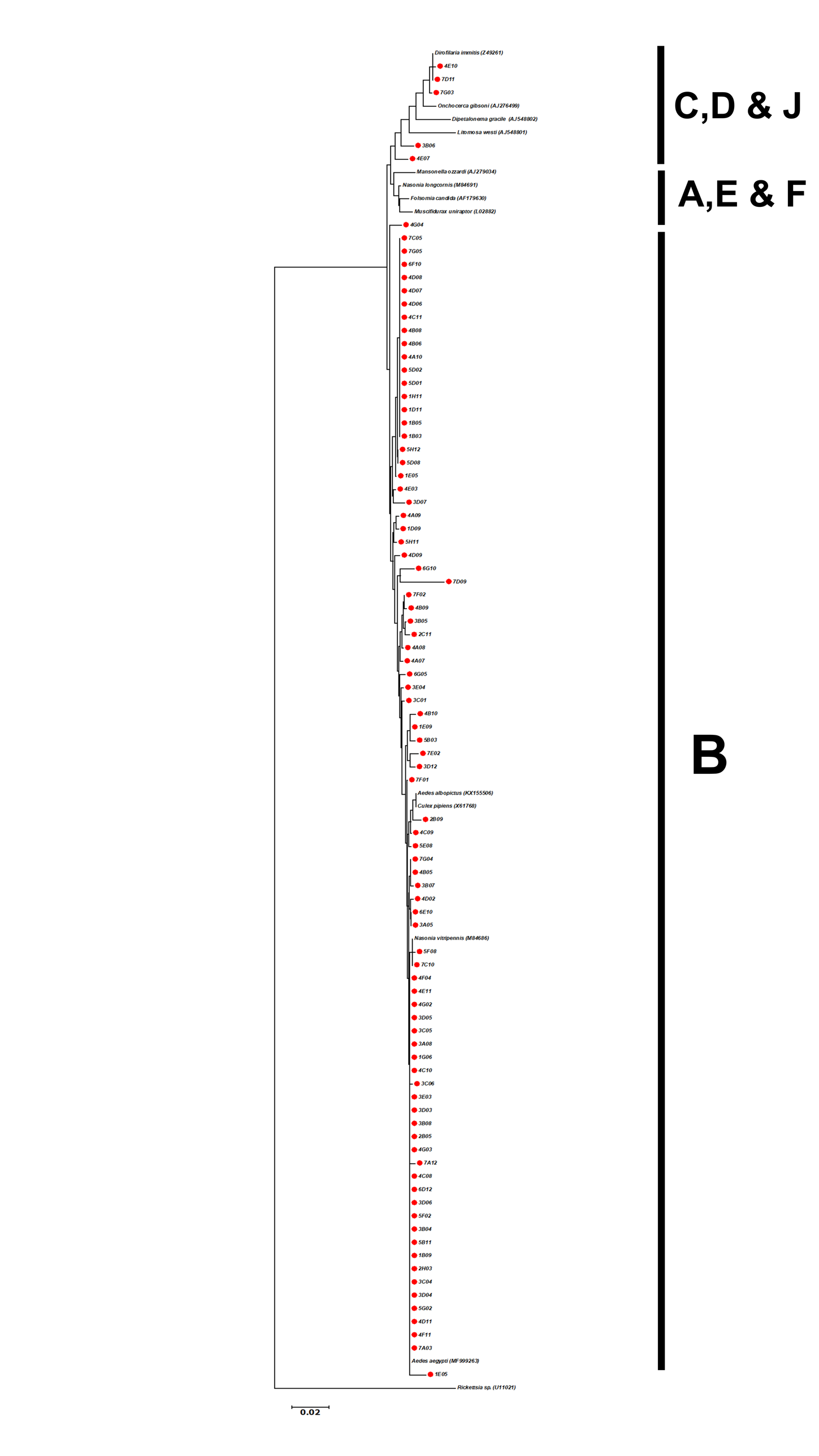

Supplement: Supplementary file 3 — Additional file 3: Figure S2. Complete 16S rDNA phylogeny of Wolbachia from Ae. aegypti (n = 85). The alignment was analyzed in the program PHYML and Rickettsia sp. was selected as an outgroup. All sample sequences are indicated as red dots. The condensed version of this tree is presented in Fig. 3. [file 13071_2019_3629_MOESM3_ESM.tif]
